# Perception of Human-Derived Risk Influences Choice at Top of the Food Chain

**DOI:** 10.1371/journal.pone.0082738

**Published:** 2013-12-18

**Authors:** Bogdan Cristescu, Gordon B. Stenhouse, Mark S. Boyce

**Affiliations:** 1 Department of Biological Sciences, University of Alberta, Edmonton, Alberta, Canada; 2 Grizzly Bear Program, Foothills Research Institute, Hinton, Alberta, Canada; University of Tasmania, Australia

## Abstract

On human-used landscapes, animal behavior is a trade-off between maximizing fitness and minimizing human-derived risk. Understanding risk perception in wildlife can allow mitigation of anthropogenic risk, with benefits to long-term animal fitness. Areas where animals choose to rest should minimize risk from predators, which for large carnivores typically equate to humans. We hypothesize that high human activity leads to selection for habitat security, whereas low activity enables trading security for forage. We investigated selection of resting (bedding) sites by GPS radiocollared adult grizzly bears (*n* = 10) in a low density population on a multiple-use landscape in Canada. We compared security and foods at resting and random locations while accounting for land use, season, and time of day. On reclaimed mines with low human access, bears selected high horizontal cover far from trails, but did not avoid open (herbaceous) areas, resting primarily at night. In protected areas bears also bedded at night, in areas with berry shrubs and *Hedysarum* spp., with horizontal cover selected in the summer, during high human access. On public lands with substantial human recreation, bears bedded at day, selected resting sites with high horizontal cover in the summer and habitat edges, with bedding associated with herbaceous foods. These spatial and temporal patterns of selection suggest that bears perceive human-related risk differentially in relation to human activity level, season and time of day, and employ a security-food trade-off strategy. Although grizzly bears are presently not hunted in Alberta, their perceived risks associated with humans influence resting-site selection.

## Introduction

Where wildlife and humans coexist, animals can modify their behavior compared to areas without human use, and anticipating these behavioral changes can benefit wildlife conservation [1]–[3]. Proactive understanding of animal behavioral response to humans is important particularly for large carnivore species sharing landscapes with human populations, because the major cause of mortality in many carnivores is conflict with people [4]–[6]. Carnivore response to human activity can be seen as analogous with prey response to predation risk [7] or spatial dynamics within predator guilds. During periods of wolf presence, elk (*Cervus elaphus*) use steeper slopes and have greater sinuousity in movements [8] whereas African ungulates avoid habitats where they are likely to be depredated [9]. Cougars (*Puma concolor*) avoid typical use areas during periods of wolf (*Canis lupus*) use [10] and the most reproductively successful female cheetahs (*Acinonyx jubatus*) are found near areas with low lion (*Panthera leo*) and spotted hyaena (*Crocuta crocuta*) densities [11].

Fear of predation has thus led to the evolution of antipredator behaviour that incorporates knowledge of environmental features (i.e., habitat characteristics) into strategies for coping with predation risk [12]. For example, prey species can reduce perceived risk and fear through evolving adaptive behaviors [13] such as choosing resting sites that offer cover (*sensu* [14]) thus minimizing the risk of detection. To minimize risk, resting sites often are located in sheltered areas, such as roe deer (*Capreolus capreolus*) fawns bedding in forest patches [15] and elk resting in low-use wolf areas [16]. Shifting habitats to densely forested areas may decrease risk of predation by cursorial predators but increase vulnerability to stalking predators [17]. Dense cover might thus have an opposite effect from that desired by the prey, by decreasing detectability of an approaching predator [18] and may be particularly ineffective at eluding olfactory predators [19]. An additional complication is that selecting areas with perceived low predation risk, while reducing direct effects, can in turn have detrimental consequences to fitness through an increase in risk effects, such as by sacrificing the amount of time spent in food-rich areas [20]. Still, because risk effects carry less cost than direct predation [20], selection for low predation risk is employed to maximize survival, but a risk-reward trade-off is likely operating in animal decision making.

When asleep, animals may have decreased ability to use evolutionary mechanisms of coping with risky situations, such as long-distance perception of danger through scent, sight or hearing, flight response, dominance displays or aggressive physical contact. Selection of resting sites is therefore an essential determinant of predation risk during the sleep period [21], and studying resting site choice can provide insights into risk perception by wild animals, possibly including carnivores. Indeed, security (horizontal cover) appears to be a key component in choice of resting sites by carnivores such as Eurasian lynx [22] and Florida panther [23], and high vertical cover is an excellent predictor of fisher resting-site selection [24], [25]. Although resting carnivores may not always be sleeping [26], choice of a secure bedding site could minimize the risk and associated costs of fleeing [27], maximizing survival probability.

Perceived predation risk from humans might be higher for unpredictable human activity occurring at irregular time intervals, such as recreation [28], although human use of trails could be more predictable than random use of the landscape [29]. Seasonally high levels of recreation (summer) may thus elicit differences in carnivore response to humans. At a finer temporal scale and particularly when displacement may not be an option because of habitat limitations or territoriality mechanisms, periods when humans are most active (day-time) may coincide with periods of low carnivore mobility/resting [30], [31] with secure habitat influencing resting-site selection [27]. Developing predictive models that possibly correlate with animal fear can improve understanding of carnivore and other wildlife response to human activity [32].

In addition to the detrimental effects of fear associated with predation risk, such as decrease in use of areas with adequate food sources, fitness also is influenced by ability to thermoregulate. Physiological comfort factors into resting-site selection [27], [33] but is difficult to monitor in field studies of wide ranging carnivores. However, canopy (hereafter, vertical cover) provides overhead thermal cover [34] and insulation from atmospheric precipitation or direct sunlight. Because wind hitting an animal's body surface decreases bodily temperature through convective heat loss [35], visibility (hereafter, horizontal cover) at resting sites may affect thermoregulation, because sites with low visibility have surrounding habitat structures that provide wind shelter.

While accounting for the potential influence of thermal comfort, this study tested whether food, security or a combination of food and security determine choice of a facultative carnivore's resting (bedding) sites, and whether differences in risk perception (fear) result in selection of sites with different types of security and food features as a function of land use, season, and time of day. We used grizzly bears (*Ursus arctos*) in a complex landscape with different levels of human activity as a study system and defined 'bed' to be a spot where a bear rested, curling up on the substrate and leaving body prints or other discernible signs [36]. We focused exclusively on beds used during the bear active season, i.e. outside bear winter denning. Our study organism is the largest North American terrestrial facultative carnivore that at its adult stage has no natural enemy except humans, or in some cases conspecifics, such as infanticidal males attacking females with cubs of the year. As the least resilient large carnivore of the Rocky Mountains [37], the grizzly bear has experienced a substantial range decline as a result of persecution by humans and habitat loss [38]. In Alberta, Canada where this study was conducted the species was designated as Threatened in 2010 because of low population estimates for the province with more than 90% of grizzly bear mortalities on record being human-caused [39]. Human access is an important predictor in models describing relative mortality risk of Alberta grizzly bears [40].

European brown bears respond to increase in risk of mortality during the legal hunting season by selecting areas with dense cover [27]. In Alberta, grizzly bear hunting is no longer allowed but human activity is on the rise in bear habitat and includes open-pit mining, logging, oil and gas development and recreation [40]. Recreation is the most unpredictable of these activities and supported by an extensive network of unpaved roads and trails that facilitate human access in bear habitat on Crown (public) lands [41]. In contrast, on reclaimed open-pit mines human access is restricted to a few designated trails, whereas in protected areas human access also is minimal.

Based on this variation in human access by land designation, we predict that in choosing resting sites grizzly bears perceive protected areas and reclaimed mines as secure because of low human use of these areas. Given the low presence of humans as well as high energy gain requirements to sustain a large body mass, we predict that the primary driver of resting-site selection in protected areas and on reclaimed mines is food. In contrast, we predict that when on Crown lands where there is high human access, bears will select areas far from people, with high cover, steep slopes and close to edge. Seasonally, we predict that bears will seek more concealment during summer because of high levels of human recreation but not during fall because human access is lower and restricted primarily to hunters, and no hunting of grizzly bears is allowed in Alberta. In regards to time of day, we expect that bears seek more cover while resting during day-time when humans are active on the landscape.

## Methods

### Study Area

**Figure 1:**
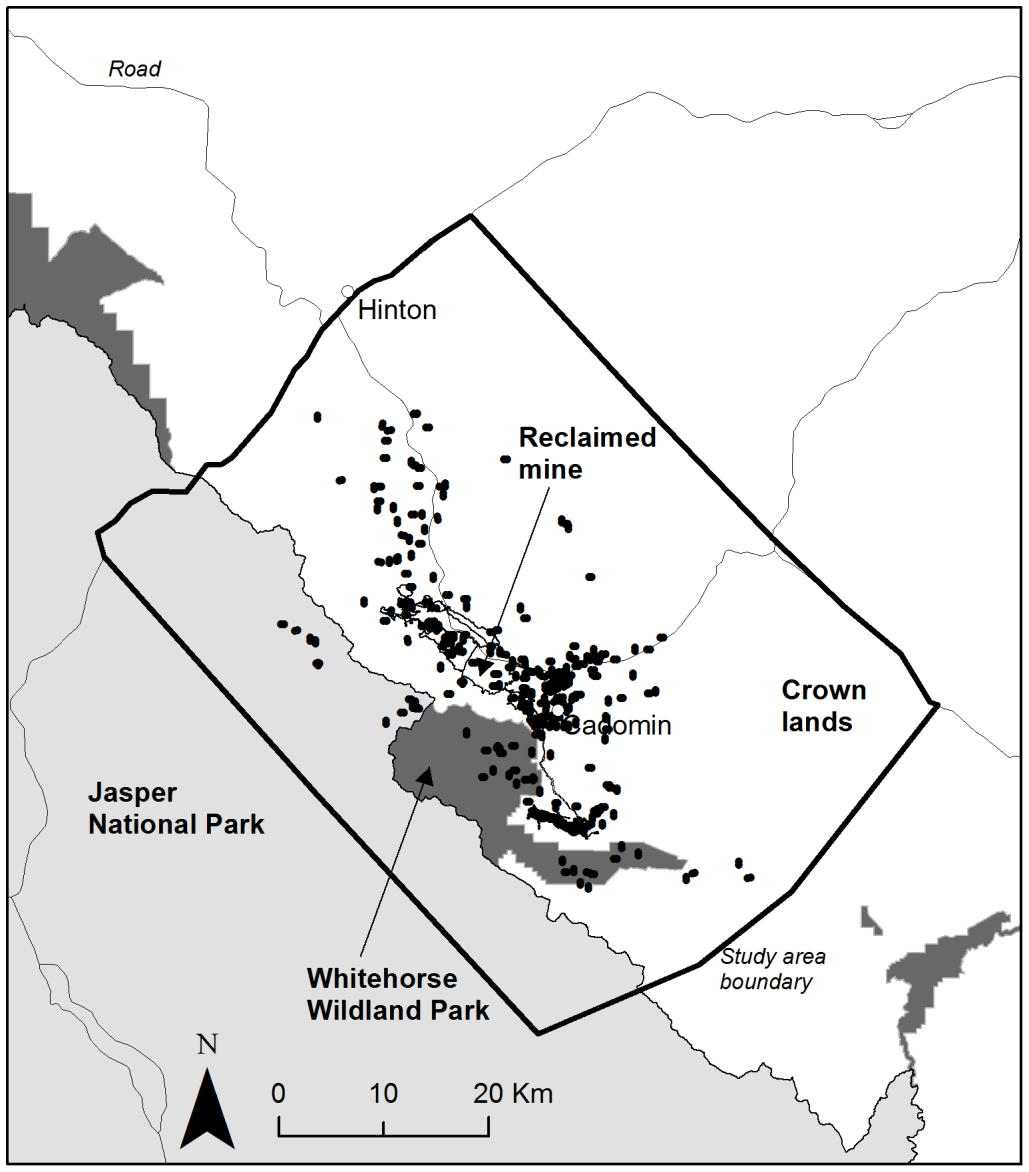
Study area for grizzly bear resting behavior in relation to perceived human-derived risk in west-central Alberta, Canada, including major roads and color coded land designations: reclaimed mines and Crown lands (white), protected areas (National park, dark gray; Wildland Park, light gray). Black dots are grizzly bear bedding (*n_1_* = 279) and paired random (*n_2_* = 279) sites 300 m away from bedding sites, visited in the field in 2009 and 2010.

The study took place in a 3,200 km^2^ area that included Rocky Mountains and foothills of west-central Alberta, Canada (Figure 1). The predominant land cover is coniferous forest composed of spruce (*Picea* spp.), fir (*Abies* spp.) and lodgepole pine (*Pinus contorta*). Mixed conifer-deciduous and deciduous forest types occur at lower elevations, being composed primarily of aspen and poplar (*Populus* spp.). No salmon is available to grizzly bears, and the main bear foods are moose (*Alces alces*), elk (*Cervus elaphus*), deer (*Odocoileus virginianus* and *O. hemionus*), bighorn sheep (*Ovis canadensis*), sweet vetch roots (*Hedysarum* spp.), herbaceous material, and berries [42], [43]. Livestock were absent from the study area and no significant human foods were available to bears.

Land use on Crown land is diverse and consists of coal mining with open pits, forestry, oil and gas exploitation, and recreational activities (All Terrain Vehicles, dirt and mountain biking, horseback riding, hiking, and hunting). The northern area boundary is a paved highway, and human access along linear features (roads and trails) is extensive on Crown lands. Two neighbouring reclaimed mines are located at the centre of the study area, one completely and the second largely reclaimed. Protected areas include Whitehorse Wildland Park and Jasper National Park. Cadomin (population = 60) is the only permanently settled community.

### Ethics Statement

Adult grizzly bears were captured and radiocollared primarily in 2009 and 2010 with assistance from the Foothills Research Institute Grizzly Bear Program (Hinton, Alberta, Canada), using the Program's capture, handling and sampling protocol. An additional bear had been captured in 2008 using the same protocol. The protocol and progress reports were annually reviewed and approved by the University of Alberta Animal Care and Use Committee for Biosciences (558804). To reduce risk of muscle injury, capture methods included culvert trapping, helicopter darting and to a lesser extent leg-hold snaring [44].

### Data Collection

Capture efforts covered Crown land, mine sites, and Whitehorse Wildland Park to avoid capture-induced bias by method or land category. Remotely downloadable GPS radiocollars (Telus UHF; Followit, Lindesberg, Sweden) were programmed to acquire a location every hour during the bear-active season (March 15-December 1). 20% of monitored bears did not have GPS radiocollar locations on reclaimed mines, 30% did not have locations in protected areas, and all bears had locations on Crown lands.

Each bear was approached monthly on the ground or from aircraft to download radiocollar data remotely, while maintaining >200 m distance to minimize disturbance. Field visitation occurred for a sample of GPS location clusters (≥3 GPS fixes), as identified with a clustering algorithm modified from [45] to include 1-h GPS fixes and 50 m Euclidean distance between the two original cluster points. An attempt was made to visit the largest four clusters for each bear during each month and randomly other clusters, thereby keeping sampling effort consistent between individual bears. Cluster sites were located based on centroids included in the algorithm output, which were transferred to hand-held GPS units. Crews accessed sites on foot or via truck, ATV or helicopter and searched a 50 m radius from the centroid for all evidence of bear activity fitting the cluster date. Visitation occurred 41±15 days after the first fix to eliminate disturbance to the animal, and because of logistical constraints.

A bed-site consisted of a depression excavated by the bear or a resting event occurring on a natural substrate contour. Even when excavations were located, only sites at which we confirmed the presence of multiple bear hairs in the bed and/or attached to the bark/branches of overhanging/adjacent tree(s) were included in the resting site analyses. Often such sites had multiple bear scats. Once the bed was located, a 20×20 m plot was delineated, centred on the bed-site (or on a randomly chosen bed if >1 beds were detected). A comprehensive habitat survey was performed at each plot where bed(s) were present as described below, and sampling was replicated at a plot 300 m away on one of 4 cardinal directions from the confirmed resting site, with clockwise choice of subsequent directions (i.e., N, E, S and W). Code names assigned to variables are italicized in brackets.

Elevation (*Elevation*) was recorded from a barometric altimeter on the GPS unit and slope (*Slope*) and aspect (*Aspect*) were recorded with a compass equipped with a clinometer. We assigned a habitat class to each site including barren land (*Barren*: <5% vegetation), herbaceous (*Herbaceous*: >5% vegetation, <5% shrub, <5% tree), shrub (*Shrub*: >5% shrub, <5% tree), mixed forest (*Mixed forest*: 21–79% coniferous forest), open conifer (*Open conifer*: >80% coniferous forest, 5–30% canopy cover), moderate conifer (*Moderate conifer*: >80% coniferous forest, 31–69% canopy cover), or dense conifer (*Dense conifer*: >80% coniferous forest, >70% canopy cover). Nine plots in regenerating coniferous forest were reclassified as open, moderate or dense conifer based on vertical cover. Canopy cover (*V cover*) was recorded using a spherical densiometer [46], taking the mean of 4 readings (N, E, S and W) at each of 5 0.7×0.7 m quadrants on a N-S transect through the plot centre. We noted vertical cover for the bed-site and overall plot average. Horizontal cover (*H cover*) at the bed was estimated using a sheet with two 50×30.48 cm red and white rectangles, modifying the method in [47]. Two readings were taken from a 10 m distance from the plot centre (N and S), while holding the sheet vertically. A 2-m factor prism was used at treed plots to derive stand basal area (*Basal area*). Forest age (*F age*) was assessed visually as immature, mature or old. Using a diameter-breast-height (dbh) tape we measured the diameter (cm) of the two largest trees in the plot and the diameter of the largest tree on a radius of 1.5 m from the bed.

Species-specific percentages cover were recorded for forbs and legumes consumed by grizzly bears [42], [48], whereas cover recording for monocots was carried out for pooled species. Recordings occurred in the 5 0.7×0.7 m quadrants along the N-S transect. Data were later converted to presence-absence (*Forbs*) because of plant phenology differences during the sampling period. Because of their omni-presence (90% of sites), monocots were excluded from analyses. The presence/absence of major berry shrubs (*Berries*) used by grizzly bears [48], [49] also was recorded using the same quadrants. Species-specific presence/absence data also was obtained for ungulates (*Ungulates*) based on search for pellets in the 20×20 m plot, but data were pooled across species because of increased model fit. When snow was present, we still recorded ground vegetation after clearing the snow from the 5 quadrants.

Based on 2-m resolution color ortho-rectified photos and field knowledge, we used ArcMap v.9.2 (ESRI, Redlands, California) to measure the distance from the plot centre to the nearest road (*Dist road*), trail (*Dist trail*) and habitat edge, defined as the border between two habitat classes as classified above (*Dist edge*). Although vertical cover, horizontal cover and elevation recorded at a site all drive the site's microclimate, we further accounted for physiological requirements of a resting bear by calculating a site-severity index (*SSI*) [50] modified from the Beer's transformation of aspect [51]. The index includes aspect and slope and provided a measure of solar insulation and moisture. Northeast slopes have low solar insulation and high moisture, corresponding to a low index, whereas southwest slopes have high solar insulation and are dry, corresponding to a high index.

### Modeling Grizzly Bear Resting Habitat

Because of sample size-caused model convergence issues when attempting to perform analyses separately for each bear gender, all statistical analyses involved pooled male and female data. Analyses are therefore representative of grizzly bear population-level resting site characteristics, with a bias towards females due to 3.8 fold larger sample size of recorded bedding events compared to males.

#### Resting-Site Selection

We applied conditional logistic regression in a discrete choice modeling framework [52] to identify variables affecting selection of resting sites at the scale of bear mean hourly step length.

The matched-case design contrasted variables recorded at each resting site with those recorded at a paired random site. The random location (0) conditionally occurred 300 m away from the resting site (1), based on knowledge of movement rates for adult bears in our study system (average step length 269 m/h excluding winter, range 175–367, data from 11 bears tracked in the area prior to this study). Even though use-availability designs are often appropriate in wildlife habitat studies [53], a matched-case design [54] was more adequate in our study. The design was uncontaminated because we found no instance of bear resting at any paired site sampled away from cluster sites.

Because we were interested in differences in bear resting-site selection in areas with different levels of human activity, we performed separate analyses for each land designation defined as: 1. reclaimed mine with minimal recreational access restricted to designated trails; 2. protected area with minimal human use (Whitehorse Wildland Park and Jasper National Park); 3. public land with high levels of recreation (Crown lands). We created three sets of *a priori* resting-site selection models for each land designation based on our understanding of bear biology and hypothesized bear response to human activity. The first set included food models with forage covariates exclusively, the second set included models for grizzly bear perceived security with no food covariates, and the final set included models that combined food and perceived security covariates. We eliminated correlated variables (Pearson |*r*| >0.6) from candidate models and assessed potential collinearity between linear covariates using variance inflation factors (VIF). Variables with individual variance inflation values >10 or the average of all values substantially larger than 1 were collinear, and therefore not included in the same model [55]. We thus eliminated forest age, stand basal area, and habitat class because of high correlations with vertical cover. We performed a distinct conditional logistic regression analysis to parameterize selection for each habitat class.

We assessed how squared terms influenced model performance and included such terms for distance to edge, road, and trail. Distance to edge is typically considered a food variable for ungulate distribution modeling, but we included it as a security variable in candidate models for grizzly bear resting-site selection because edges serve security functions for this species [56]. Robust standard errors were computed to control for heteroskedasticity and minimize bias in parameter estimation for all models.

We used ΔAICc (small sample size correction for AIC) and AICc weights to determine top models [57], [58] for each land designation and every model set (food, security, food + security). The top three models (four if the weights for two models were identical) from each set were included in a second and similar model selection procedure which ranked competing food, security, and food + security models. Following [59], we used Area Under the Curve (AUC) to assess the predictive power of top models, and sensitivity and specificity to derive the optimal probability cut-off for assigning presence/absence of a resting site [60].

#### Influence of Non-Habitat Factors on Resting-Site Selection

We identified three non-habitat related factors that could influence patterns of resting-site selection by bears: land designation, season and time of day. Land designation followed the classification described above. Based on [56] and our first and last field confirmed bedding event, we divided the data into three seasons: “hypophagia” (spring; April 21 to June 14), “early hyperphagia” (summer; June 15 to August 7) and “late hyperphagia” (fall; August 8 to October 28), and pooled data across the two years of monitoring. Time of day (period) when resting commenced was classified as diurnal (sunrise to sunset), crepuscular (morning twilight to sunrise and sunset to evening twilight) and nocturnal (evening twilight to morning twilight) time periods. We used sunrise, sunset and civil twilight tables (http://www.cmpsolv.com/los/sunset.html, accessed October 17, 2011) based on expected conditions for the centre of our study area (Cadomin, Alberta, Canada; 53°N, 117°20'W) in the Mountain Time zone. We assessed whether bears rested more at certain times of day by performing chi-square tests for each land designation.

We used generalized linear models (GLM; Gaussian family) with maximum likelihood optimization to investigate the effects of the three factors on all four variables that significantly influenced resting-site selection, as identified from the resting-site selection models: vertical and horizontal cover, distance to edge and distance to trail. We included the three non-habitat factors and interaction terms in candidate models following calculation of Pearson correlation coefficients between predictor variables and VIF diagnostics at above specified cut-offs. To incorporate habitat availability, dependent variables were inputted as ratios calculated by dividing the habitat value at each resting site by the value at each associated random site. For example, the value for vertical cover on top of a given bed-site was divided by the value for vertical cover at the paired random site. Prior to inclusion in models, all dependent variables were log-transformed to obtain Gaussian distributions. The model took the form
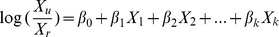



where 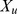 was the value for the habitat variable of interest (e.g., vertical cover) recorded at the resting site, 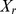 was the value for the same habitat variable at the paired random site, 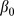 was the intercept, and 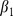 to 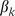 were estimated GLM coefficients for predictor variables 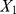 to 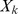. We used robust standard errors to further control for imperfect normality and heteroskedasticity. We ranked candidate models using ΔAICc and AICc weights which allowed identification of the top model for each of the dependent variable investigated. For all four best models we plotted standardized Pearson and deviance residuals, inspected the residuals for normality and used the Pregibon leverage statistic [61] to identify potential observations that influenced coefficient sensitivity. We re-ran the models without these observations and checked for differences in coefficient estimation. Individual observations and combinations of these had little influence on regression output.

#### Within-plot Resting-Site Selection

By within-plot selection we refer to selection of habitat features at the micro-scale (within the 20×20-m field-delineated plot). For all sites where resting was confirmed by field visitation, we compared mean vertical cover on top of the bed with mean vertical cover for the plot using Wilcoxon matched-pairs signed-ranks tests as the data did not follow a Gaussian distribution.

## Results

In 2009 and 2010, we captured and deployed GPS radiocollars on 12 adult grizzly bears in the study area. Two large males dropped the collars within a month of capture and were not considered for analyses. The remaining bears included 6 females and 4 males we monitored for 383 bear-days during hypophagia (mean 7.1 bears), 683 bear-days in early hyperphagia (mean 12.6 bears) and 640 bear-days in late hyperphagia (mean 7.8 bears). During May-November 2009 and 2010 we located a total of 279 bedding sites 19% of which were found on reclaimed mines, 14% in protected areas and 67% on Crown lands (Table S1 in File S1). Additional sites (*n* = 66) with confirmed bear beds were excluded from analyses because they were located in a buffer area between reclaimed mines and public land (mineral surface leases outside reclamation) which did not fit our land categorization. The 50 m radius search performed at each GPS location cluster revealed that regardless of land designation most sites had a single bear bed; reclaimed mine had the highest proportion of single beds (1.04±0.19 beds/site) followed by protected areas (1.21±0.57 beds/site) and Crown lands (1.28±0.72 beds/site).

### Resting-Site Selection

**Figure 2:**
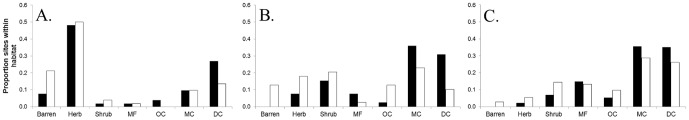
Habitat class at 279 grizzly bear resting sites and 279 random sites in west-central Alberta, Canada, by land designation: A. Reclaimed mines (*n_1_* = 52 bedding sites), B. protected areas (*n_2_* = 39 bedding sites) and C. Crown lands (*n_3_*  = 188 bedding sites). Classification includes barren land (Barren), herbaceous (Herb), shrub (Shrub), mixed forest (MF), open conifer forest (OC), moderate conifer forest (MC) and dense conifer forest (DC).

Bears avoided barren land for resting regardless of land designation (Table 1; Figure 2). When on reclaimed mines, bears selected open conifer forest for resting, a habitat class little represented on mines and composed of regenerating conifer trees. When in protected areas and Crown lands, bears had negative selection for resting in the herbaceous land class. The strongest response to habitat class was on Crown lands, where bears not only avoided open habitats (barren and herbaceous) when choosing resting sites, but also selected against more concealed habitats such as open conifer and shrub, when compared to dense conifer. As shown by the goodness-of-fit Wald chi-square test results, the habitat class models were significant and with good predictive power (reclaimed mines: AUC = 0.73; protected areas: AUC = 0.87) except for the Crown land model (AUC = 0.68). Optimal probability cut-offs were 50% for all three models.

**Table 1:** Estimated coefficients (*β_i_*), robust standard errors [SE] and 95% confidence intervals [CI] for categorical habitat models describing the probability of occurrence for grizzly bear resting sites by land designation in west-central Alberta, Canada.

		Reclaimed mine				Protected area				Crown land			
Variable		*β_i_*	Robust SE	95% CI		*β_i_*	Robust SE	95% CI		*β_i_*	Robust SE	95% CI	
*Habitat class*													
	Barren	−2.794	1.054	−4.862	−0.727	−34.792	1.254	−37.250	−32.334	−15.837	0.601	−17.015	−14.660
	Herbaceous	−0.687	0.647	−1.956	0.582	−18.195	1.029	−20.213	−16.178	−1.720	0.679	−3.051	−0.390
	Shrub	−2.082	1.738	−5.489	1.325	−1.845	1.141	−4.083	0.392	−1.369	0.467	−2.285	−0.454
	Mixed forest	−0.343	1.263	−2.818	2.132	−0.461	1.336	−3.080	2.157	−0.511	0.448	−1.389	0.367
	Open conifer	15.717	0.964	13.829	17.606	−1.889	1.609	−5.043	1.264	−1.332	0.528	−2.368	−0.296
	Moderate conifer	−1.389	1.221	−3.783	1.005	−0.341	0.794	−1.897	1.216	−0.310	0.291	−0.880	0.260
*Model eval.*		?^2^	df	*P*		?^2^	df	*P*		?^2^	df	*P*	
	Wald test	535.7	6	<0.0001		1516.8	6	<0.0001		905.8	6	<0.0001	
	ROC (AUC)	0.73				0.87				0.68			
	Cut-off probab.	0.5				0.5				0.5			

[CI] did not overlap zero are given in bold. Estimates for which the

Dense conifer was withheld as a reference category.

Of the 36 candidate models tested for each land designation (9 food models; 14 security models; 13 combined food and security models), only the top model for reclaimed mines had substantial support (Δ*_i_*<2) (Table S2 in File S1). In comparison, three models for protected areas (Table S3 in File S1) and three models for Crown lands (Table S4 in File S1) received substantial support. Security and combined food and security models had varying amount of support whereas food models had essentially no support (Δ*_i_*>10). The top model for reclaimed mines (*w_i_* = 0.49) was a model with security variables only. The top model for protected areas (*w_i_* = 0.37) was a combined food and security model, with the second and third ranked models having security variables only. The top (*w_i_* = 0.40) model for Crown land also was a combined food and security model, whereas the second and third ranked models included security variables only. Goodness-of-fit Wald chi-square tests revealed that all top bedding site selection models had good model fit at an alpha 0.01 level of significance (Table 2). The percentage deviance explained varied between the different models, with the largest amount of deviance explained by the best models for resting on reclaimed mines (42.9%) and protected areas (43.5%) whereas the Crown land model accounted for 19.1% of the deviance. The models for reclaimed mines and protected areas had high predictive power (reclaimed mine: AUC = 0.91; protected area: AUC = 0.90) and the Crown land model had good predictive power (AUC = 0.78). For all top models, optimal probability cut-offs were close to 50%.

**Table 2:** Estimated coefficients (*β_i_*), robust standard errors [SE] and 95% confidence intervals [CI] for top models describing the probability of occurrence for grizzly bear resting sites by land designation in west-central Alberta, Canada as assessed by Δ*_i_* and *w_i_*.

		Reclaimed mine				Protected area				Crown land			
Variable		*β_i_*	Robust SE	95% CI		*β_i_*	Robust SE	95% CI		*β_i_*	Robust SE	95% CI	
*Forage*													
	Forbs									0.659	0.373	−0.071	1.389
	Berries					2.512	2.005	−1.418	6.441				
*Security*													
	V cover	−0.163	0.104	−0.037	0.004	**0.046**	**0.014**	**0.019**	**0.074**	**0.025**	**0.006**	**0.014**	**0.036**
	H cover	**0.869**	**0.329**	**0.224**	**1.514**	0.166	0.182	−0.190	0.522	**0.208**	**0.102**	**0.008**	**0.409**
	Slope												
	Slope^2^												
	Dist edge	−0.022	0.016	−0.054	0.010					**−0.019**	**0.006**	**−0.032**	**−0.007**
	Dist edge^2^	−0.022^∧^	0.081∧	−0.181∧	0.137∧					**0.065∧**	**0.022∧**	**0.023∧**	**0.108∧**
	Dist trail	**0.103**	**0.004**	**0.002**	**0.019**								
	Dist trail^2^	−0.003∧	0.006∧	−0.181∧	0.137∧								
*Comfort*													
	SSI	0.859	1.062	−1.223	2.941	1.649	0.922	−0.158	3.457	0.028	0.485	−0.923	0.979
*Model eval.*		?^2^	df	*P*		?^2^	df	*P*		?^2^	df	*P*	
	Wald test	20.5	7	0.005		14.6	4	0.006		37.9	6	<0.0001	
	ROC (AUC)	0.91				0.90				0.78			
	Cut-off probab.	0.508				0.501				0.500			

[CI] did not overlap zero are given in bold. Missing estimates for habitat features refer to variables not present in the respective model. Estimates for which the

∧ Coefficient reported at 10^3^ times its actual value.

**Figure 3:**
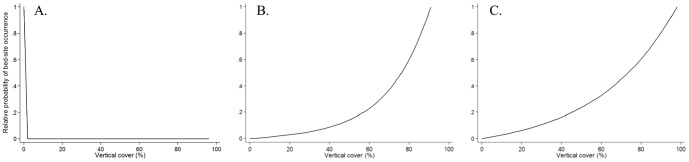
Relative probability of occurrence from AIC_c_-selected grizzly bear resting-site selection models on reclaimed mines (A), protected areas (B), and Crown lands (C) in west-central Alberta, Canada, given vertical cover.

**Figure 4:**
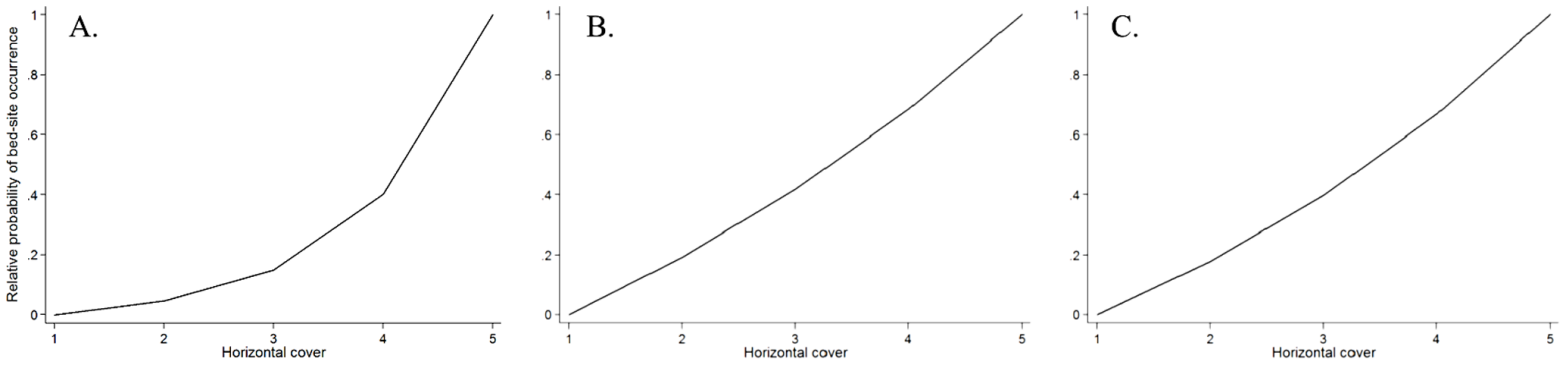
Relative probability of occurrence from AIC_c_-selected grizzly bear resting-site selection models on reclaimed mines (A), protected areas (B), and Crown lands (C) in west-central Alberta, Canada, given horizontal cover.

The top models were complex for reclaimed mines (*K_i_* = 8) and Crown lands (*K_i_* = 7) whereas the protected-area top model had an intermediate number of parameters (*K_i_* = 5) (Table 2). Irrespective of land designation, vertical cover and, with one exception, horizontal cover, were present in all models that received substantial support. Bears selected areas with high vertical cover when resting in protected areas and on Crown land but did not select vertical cover for bedding on reclaimed mines (Figure 3). When resting on reclaimed mines and Crown lands, they selected strongly for high horizontal cover but there was no strong selection when in protected areas (Figure 4). Distance to edge was a variable in all models for reclaimed mines and Crown land that received substantial support but was absent from all best models for protected areas. Bears selected areas close to edge when bedding on Crown land. When selecting resting sites on reclaimed mines, bears avoided areas near human access trails but distance to trail was absent from best models for protected areas and Crown lands. Model ranking is provided in supplementary material for reclaimed mines (Table S5 in File S1), protected areas (Table S6 in File S1) and Crown lands (Table S7 in File S1).

### Influence of Non-habitat Factors on Resting-Site Selection

**Figure 5:**
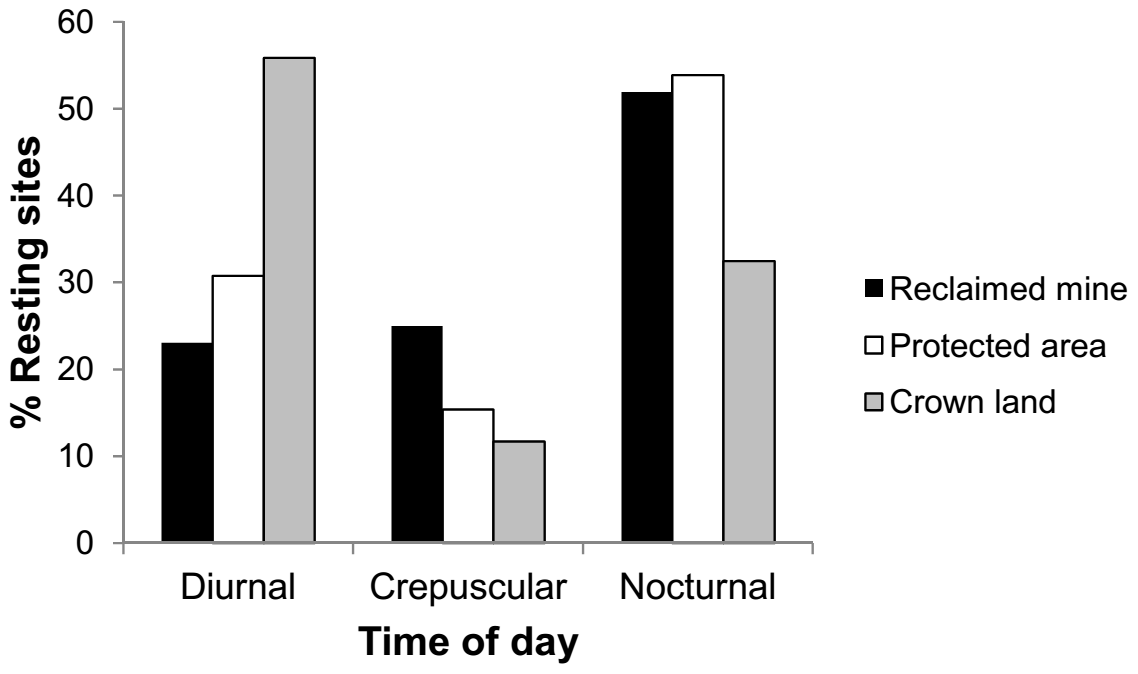
Onset time of grizzly bear resting in west-central Alberta, Canada by land designation: reclaimed mines (*n_1_* = 52 bedding sites), protected areas (*n_2_* = 39 bedding sites) and Crown lands (*n_3_*  = 188 bedding sites). Time of bedding includes diurnal (sunrise to sunset), crepuscular (morning twilight to sunrise and sunset to evening twilight) and nocturnal (evening twilight to morning twilight).

When on reclaimed mines, bears were most likely to rest at night and relatively equally likely to rest during the day and at crepuscular times (χ^2^ = 8.1, *df* = 2, *P* = 0.017) (Figure 5). In protected areas, bears were most likely to rest at night and least likely to rest during crepuscular times (χ^2^ = 8.8, *df* = 2, *P* = 0.012). On Crown lands, bears were most likely to rest during the day and least likely to rest during crepuscular times (χ^2^ = 55.04, *df* = 2, *P*<0.0001).

Our variable combinations for season, land designation and time of day were adequate at explaining selection ratios for bear resting. The best models of a suite of 10 candidate models ranked for each of four selection ratios are presented in Table S8 in File S1. The models for vertical and horizontal cover selection ratios were the only ones for which the coefficients did not overlap zero, therefore we report the estimates from the top models for these two factors only (Table 3), with estimates for all cover models reported in Table S9 in File S1. Of the 10 candidate models that influenced selection of vertical and horizontal canopy cover respectively, only the top candidate models had substantial support (Δ*_i_*>2) while the remaining models had no support (Δ*_i_*>10). The top models had a disproportionate weight of evidence compared to the competing models (vertical cover model: *w_i_* = 1.00; horizontal cover model: *w_i_* = 0.99). Both models were complex (*K_i_* = 4) and included the same set of parameters which were land designation, season and an interaction term between land designation and season. Bears selected against vertical cover when choosing resting sites in protected areas in the fall. They also selected sites with more horizontal cover when resting in protected areas and on Crown lands during summer.

**Table 3:** Estimated coefficients (*β_i_*), robust standard errors [SE] and 95% confidence intervals [CI] for top models describing log selection ratios for vertical (V) and horizontal (H) cover at grizzly bear resting sites in west-central Alberta, Canada as assessed by Δ*_i_* and *w_i_*.

		V cover				H cover			
Variable		*β_i_*	Robust SE	95% CI		*β_i_*	Robust SE	95% CI	
*Land design.*									
	Protected	1.806	1.564	−1.259	4.871	−0.154	0.327	−0.794	0.487
	Crown	−1.372	1.333	−3.984	1.239	−0.524	0.276	−1.065	0.016
*Season*									
	Summer	−1.887	1.432	−4.693	0.919	−0.721	0.274	−1.258	−0.183
	Fall	2.583	1.720	−0.788	5.954	0.391	0.301	−0.199	0.981
*Time of day*									
	Nocturnal								
*Interactions*									
	Summer × Protected	0.815	2.257	−3.609	5.239	1.057	0.492	0.093	2.021
	Summer × Crown	1.763	1.492	−1.161	4.686	0.768	0.306	0.169	1.367
	Fall × Protected	−5.098	2.002	−9.021	−1.175	−0.746	0.403	−1.536	0.044
	Fall × Crown	−2.497	1.769	−5.963	0.970	−0.456	0.333	−1.110	0.198
*Constant*		2.094	1.291	−0.435	4.624	0.750	0.251	0.257	1.242

Missing estimates refer to variables not present in the respective model. Estimates for which the confidence intervals do not overlap 0 are given in bold.

The following strata within variables were withheld as reference category:

Reclaimed mine (Land designation); Spring (Season); Diurnal (Time of day).

Only two candidate models testing the influence of non-habitat factors on distance to edge selection ratio had substantial support. Both models included land designation and time of day, with the better model also including an interaction term (model with interaction: *w_i_* = 0.49; simpler model: *w_i_* = 0.37). The distance to trail selection ratio models had poor fit, with only one model having greater weight than the corresponding null model (best model: *w_i_* = 0.22; null: *w_i_* = 0.19).

### Within-plot Resting-Site Selection

Given an alpha level of 0.1, vertical cover at resting sites located on reclaimed mines differed between the actual bed and mean vertical cover for the 20×20-m plot at the centre of which the bed was located (Wilcoxon matched-pairs signed-ranks test, *z* = 1.90, *P* = 0.06). Vertical cover on top of the bed differed substantially from the mean vertical cover for the plot for protected areas (Wilcoxon matched-pairs signed-ranks test, *z* = 4.3, *P*<0.0001) and Crown lands (Wilcoxon matched-pairs signed-ranks test, *z* = 6.7, *P* <0.0001), with the higher cover on top of the bed.

## Discussion

While it is widely recognized that protecting vast areas of habitat is key for the long-term persistence of large carnivore populations, expansion of human activities into carnivore habitat increases the potential for conflict with humans [5], [62], [63]. Herein we related recreational human activities according to land designation, season and time of day to the behavioral choice of a facultative carnivore's resting habitat selection and found differential selection associated with variation in perceived human-related risk.

Grizzly bears have evolved in predominantly open environments with the large body size serving as a protection against possible aggressors [64]. However, during periods of unpredictable and intrusive human activity, for example bear hunting season, brown bears in Scandinavia select areas far from humans that also provide high concealment [27]. Such selection might be an example of adaptive trait compensation (*sensu* [65]) in which the hiding strategy in the adaptive behavior of avoiding 'predation' by hunters compensates for uselessness of morphological defences during resting. In contrast, grizzly bears in Alberta are currently protected from hunting so we did not expect strong selection for secure habitats during fall (ungulate hunting season) because human activity levels in the fall are low compared to summer. We expected variation in resting-site selection by land designation in relation to differential human access.

Although habitat on reclaimed mines was likely secure because of restricted human access, we found that bears perceived reclaimed mines as relatively insecure, selecting horizontal cover and avoiding areas close to trails for bedding. However, there was no selection against herbaceous areas (Table 1) and 48% of beds on mines were in open grasslands characteristic of reclaimed mines. Herbaceous areas on mines generally have high horizontal cover because grasses and forbs sown as part of reclamation can reach ∼1 m height at maturity. Furthermore, 52% of resting events commenced at night (Figure 5), suggesting that perceived risk while on reclaimed mines did not induce major changes in the expected normal behavioral patterns of grizzly bears (bedding at night). Our finding of bears resting in herbaceous areas contrasts with the findings for brown bears in Scandinavia [36] and an earlier study of grizzly bears in our study area [42]. In the latter study the probability of bear resting in herbaceous areas was zero. The strong avoidance of trails is surprising given the overall low levels of human access and may be indicative of negative past experiences or detection of people from far distances because of habitat openness.

Bears perceived protected areas as secure, with vertical cover being the only variable for habitat security included in the best resting-site selection model. Vertical cover may be more indicative of thermal comfort than affording security [34] and the best model included an association of resting sites with presence of berry shrubs. Although we found no influence of trails on resting-site selection, the GLM model for horizontal cover selection ratio showed that bears select horizontal cover in the summer, which is the season when human access in protected areas is the highest. Interestingly, in the fall bears select bedding sites in areas with low vertical cover. We believe that this pattern relates to the distribution of foods consumed by bears at that time of year. In areas below 1,700 m elevation *Hedysarum* spp. roots are the primary food consumed in our study area starting late September [42] and presence of roots at fall sites in protected areas had a slight negative correlation with vertical cover (*r* = −0.22). This suggests a potential trade-off of body heat loss when being exposed to fall atmospheric precipitation while resting versus energetic gain of being present where the food is and avoiding energy loss when travelling through snow. We do not think that bedding where food is present exposes bears to high risk of intra-specific competition because bear density in the area is low [66].

Although we expected avoidance of areas close to trails and roads on Crown lands, distances to these linear human access features were not included in top models. Our results suggest that roads and trails do not influence resting-site selection by bears on Crown lands, but bears select sites with high horizontal cover and close to habitat edge for resting in these high human-use areas. Alternatively, because of the widespread network of trails and roads on Crown lands avoidance of linear features by bears may be difficult, resulting in an apparent random selection of resting sites in relation to such features. In support of the latter possibility, which would mask actual avoidance of roads and trails for bedding, during travel bears in our study area move close to roads throughout the day [67]. This movement pattern contrasts with the daytime road avoidance by grizzly bears inhabiting an area of intensive resource extraction [30], and with bears in mountainous areas with limited human use having an inverse response to access compared to those in an area with high human use [68]. Although we identified a degree of association between bear resting sites on Crown lands and presence of herbaceous foods, which provides partial support to previous research showing that on public lands bears select areas close to roads to graze on plants such as clover (*Trifolium* spp.) [69], the major drivers of resting site selection were security-related variables.

Bears bedded more at daytime on Crown lands, indicating a temporal mechanism of avoiding people. Similarly, Scandinavian brown bears alter their nocturnal resting behaviour in response to perceived risk from humans, becoming more active at night [70]. However, we cannot reach the same conclusion as [71] where bears became negatively conditioned to human activity, temporally and spatially avoiding high human-use areas. A similar pattern of temporal avoidance of humans was found for grizzly bears in the Bow Valley of Alberta avoiding trails during the human active period [31]. The temporal avoidance mechanism does show that although herbaceous foods are present at bear resting sites on Crown land, this land designation is still perceived as risky, a finding which does not support the idea of habituation to people. While habituation of grizzly bears to hikers may alleviate human-bear conflict by reducing the risk of fear-induced charges [72], on Crown lands in Alberta where many trail users carry firearms during the ungulate hunting season habituation might also expose bears to increased risk of mortality through illegal shooting.

Our *a priori* expectation that bears would perceive risk differently in relation to levels of human activity was supported by the discrete choice models as well as by GLM analyses of selection ratios for four factors that promoted security, with all best models including land designation as a variable. Of the three land designations where resting sites were located, spatial avoidance of people (resting far from trails) was a factor only on reclaimed mines, whereas temporal avoidance came into play on Crown lands. Seasonal strong selection for horizontal cover during high human activity (summer and fall) corresponds to Scandinavian brown bear selection of high cover in summer and fall [27].

Previous studies have generalized the idea that resting sites are selected based on habitat security and there have been few attempts to assess the influence of food resources on where bedding events occur. We tested the effects of occurrence of major food items, cover, slope, elevation, distance to nearest road, trail and edge on bedding site selection and also incorporated a site severity index which improved model fit and accounted for physiological comfort required by bears. Although we monitored a substantial proportion of the grizzly bear population in the 3,200 km^2^ study area (*n* = 10 adult bears, in an area with a population density of 4.79 bears/1,000 km^2^ [66]), the results are based on pooled data across bear reproductive class, age and sex because of sampling limitations.

We found that although grizzly bear resting sites can be associated with the occurrence of major plant foods (berry shrubs, herbaceous forage and potentially *Hedysarum* spp. roots), food factors are not important predictors of choice of bedding sites. Our results demonstrate that vertical and horizontal cover along with distance to trail and edge are important drivers of resting-site selection in grizzly bears. Other researchers [36], [73] also found cover to be an important factor in brown and black bear bedding-site selection, respectively. Slope, elevation and distance to road had poor predictive power in our study system which is in contrast with the findings that brown [36] and black bear [73] beds occur on steep slopes, at higher elevation and far from roads, although in the above mentioned Scandinavian brown bear study sheep-killing bears did not avoid forestry roads for bedding.

In our study system there is indication that bears exhibit complex behavioral mechanisms to minimize perceived human-derived risk including selection for cover, edge, spatial avoidance of areas near trails and temporal avoidance of people for resting, depending on land designation and season. Larger sample sizes enabling analyses by reproductive class, age and sex would provide finer scale understanding of trade-offs involved in bear resting site selection in west-central Alberta and other study systems. The beginning of the bear hunting season has a remarkable effect on Scandinavian brown bears, which become more nocturnal after hunting starts [70]. Although grizzly bears are not hunted in Alberta, the moratorium on grizzly bear hunting was introduced in 2006, only three years before our data collection and four years before the species was designated as Threatened. It is therefore possible that choice of resting sites by bears may be influenced by the 'ghost of predation past' [74], a proposition that has also been put forward for brown bear reproductive allocation [75].

## Supporting Information

File S1 **Table S1.** Adult grizzly bear resting sites confirmed during field visitation of GPS radiocollar location clusters in 2010 and 2011 in west-central Alberta, Canada. **Table S2.** Model structure and deviance for top 3 resting-site selection models (RSFs) for grizzly bear resting on reclaimed mines in west-central Alberta, Canada. Model assessment was done by ranking AIC_c_ values (Δ*_i_*) and weights (*w_i_*) describing model likelihood. Model complexity (number of parameters) is given by *K_i_*. The top resting-site selection models were selected from candidate food, security and combined food and security models also selected via Δ*_i_* and *w_i_*. Only the top models from the latter categories are given below with the full set of models available in the Table S7. The best overall model is given in bold. **Table S3.** Model structure and deviance for top 3 resting-site selection models (RSFs) for grizzly bear resting in protected areas in west-central Alberta, Canada. Model assessment was done by ranking AIC_c_ values (Δ*_i_*) and weights (*w_i_*) describing model likelihood. Model complexity (number of parameters) is given by *K_i_*. The top resting-site selection models were selected from candidate food, security and combined food and security models also selected via Δ*_i_* and *w_i_*. Only the top models from the latter categories are given below with the full set of models available in Table S8. The best overall model is given in bold. **Table S4.** Model structure and deviance for top 3 resting-site selection models (RSFs) for grizzly bear resting on non-mined Crown (public) land in west-central Alberta, Canada. Model assessment was done by ranking AIC_c_ values (Δ*_i_*) and weights (*w_i_*) describing model likelihood. Model complexity (number of parameters) is given by *K_i_*. The top resting-site selection models were selected from candidate food, security and combined food and security models also selected via Δ*_i_* and *w_i_*. Only the top models from the latter categories are given below with the full set of models available in Table S9. The best overall model is given in bold. **Table S5.** Model structure and deviance for candidate models for grizzly bear resting on reclaimed mines in west-central Alberta, Canada. Model assessment was done by ranking AIC_c_ values (Δ*_i_*) and weights (*w_i_*) describing model likelihood. Model complexity (number of parameters) is given by *K_i_*. The top resting-site selection models were selected from candidate food, security and combined food and security models also selected via Δ*_i_* and *w_i_*. **Table S6.** Model structure and deviance for candidate models for grizzly bear resting in protected areas in west-central Alberta, Canada. Model assessment was done by ranking AIC_c_ values (Δ*_i_*) and weights (*w_i_*) describing model likelihood. Model complexity (number of parameters) is given by *K_i_*. The top bedding site selection models were selected from candidate food, security and combined food and security models also selected via Δ*_i_* and *w_i_*. **Table S7.** Model structure and deviance for candidate models for grizzly bear resting on non-mined Crown (public) land in west-central Alberta, Canada. Model assessment was done by ranking AIC_c_ values (Δ*_i_*) and weights (*w_i_*) describing model likelihood. Model complexity (number of parameters) is given by *K_i_*. The top bedding site selection models were selected from candidate food, security and combined food and security models also selected via Δ*_i_* and *w_i_*. **Table S8.** Model structure and deviance for top GLM models testing the influence of season, land designation and time of day on selection ratios for grizzly bear resting in west-central Alberta, Canada. Model assessment was done by ranking AIC_c_ values (Δ*_i_*) and weights (*w_i_*) describing model likelihood. Model complexity (number of parameters) is given by *K_i_*. **Table S9.** Model structure, deviance, significance and goodness-of-fit (Wald _χ_^2^) for top GLM models testing the influence of season, land designation and time of day on vertical and horizontal cover selection ratios for grizzly bear resting in west-central Alberta, Canada. Model assessment was done by ranking AIC_c_ values (Δ*_i_*) and weights (*w_i_*) describing model likelihood. Model complexity (number of parameters) is given by *K_i_*. The full set of candidate models including the null models is provided below.
(DOC)
