# Supplementary material for: Perception of Human-Derived Risk Influences Choice at Top of the Food Chain
Source: PLoS One. 2013 Dec 18;8(12):e82738. doi: 10.1371/journal.pone.0082738 (PMC3867378; doi:10.1371/journal.pone.0082738)
Supplement: File S1 — Table S1. Adult grizzly bear resting sites confirmed during field visitation of GPS radiocollar location clusters in 2010 and 2011 in west-central Alberta, Canada. Table S2. Model structure and deviance for top 3 resting-site selection models (RSFs) for grizzly bear resting on reclaimed mines in west-central Alberta, Canada. Model assessment was done by ranking AICc values (Δi) and weights (wi) describing model likelihood. Model complexity (number of parameters) is given by Ki. The top resting-site selection models were selected from candidate food, security and combined food and security models also selected via Δi and wi. Only the top models from the latter categories are given below with the full set of models available in the Table S7. The best overall model is given in bold. Table S3. Model structure and deviance for top 3 resting-site selection models (RSFs) for grizzly bear resting in protected areas in west-central Alberta, Canada. Model assessment was done by ranking AICc values (Δi) and weights (wi) describing model likelihood. Model complexity (number of parameters) is given by Ki. The top resting-site selection models were selected from candidate food, security and combined food and security models also selected via Δi and wi. Only the top models from the latter categories are given below with the full set of models available in Table S8. The best overall model is given in bold. Table S4. Model structure and deviance for top 3 resting-site selection models (RSFs) for grizzly bear resting on non-mined Crown (public) land in west-central Alberta, Canada. Model assessment was done by ranking AICc values (Δi) and weights (wi) describing model likelihood. Model complexity (number of parameters) is given by Ki. The top resting-site selection models were selected from candidate food, security and combined food and security models also selected via Δi and wi. Only the top models from the latter categories are given below with the full set of models available in [file pone.0082738.s001.doc]

**SUPPORTING INFORMATION**

**Table S1** Adult grizzly bear resting sites confirmed during field visitation of GPS radiocollar location clusters in 2010 and 2011 in west-central Alberta, Canada.

|  | **Reclaimed mine** | | | **Protected area** | | | **Crown land** | | |  |
| --- | --- | --- | --- | --- | --- | --- | --- | --- | --- | --- |
|  | Spring | Summer | Fall | Spring | Summer | Fall | Spring | Summer | Fall | Total |
| Bedding sites (#) | 10 | 37 | 5 | 9 | 4 | 26 | 47 | 64 | 77 | 279 |
| Bears | *G23* *G37* *G111* *G113* *G118* G112 G115 | | | *G23* *G37* *G111 G117* G110 G115 | | | *G23* *G37* *G111 G113 G117 G118* G53 G112 G115 | | |  |

Unique identities of male bears are in regular font and italicized for females.

**Table S2** Model structure and deviance for top 3 resting-site selection models (RSFs) for grizzly bear resting on reclaimed mines in west-central Alberta, Canada. Model assessment was done by ranking AIC_c_ values (Δ*_i_*) and weights (*w_i_*) describing model likelihood. Model complexity (number of parameters) is given by *K_i_*. The top resting-site selection models were selected from candidate food, security and combined food and security models also selected via Δ*_i_* and *w_i_*. Only the top models from the latter categories are given below with the full set of models available in the Table S7. The best overall model is given in bold.

|  | **Model** |  | ***K_i_*** | **-2LL** | **% Dev. explained** | **AIC_c_** | **Δ*_i_*** | ***w_i_*** |
| --- | --- | --- | --- | --- | --- | --- | --- | --- |
|  | Null model |  | 1 | 72.1 | 0.0 | 74.2 | 12.5 | 0.00 |
| *Forage* |  |  |  |  |  |  |  |  |
|  | Forbs + Berries + SSI |  | 4 | 64.4 | 10.7 | 73.5 | 11.8 | 0.00 |
|  | Berries + SSI |  | 3 | 67.3 | 6.6 | 74.0 | 12.3 | 0.00 |
| *Security* |  |  |  |  |  |  |  |  |
|  | **V cover + H cover + Dist trail + Dist trail^2^ + Dist edge + Dist edge^2^ + SSI** |  | **8** | **41.2** | **42.9** | **61.7** | **0.0** | **0.49** |
|  | V cover + H cover + Dist edge + Dist edge^2^ + SSI |  | 6 | 49.3 | 31.7 | 63.7 | 2.0 | 0.18 |
|  | Dist edge + Dist edge^2^ + SSI |  | 4 | 56.2 | 22.0 | 65.3 | 3.6 | 0.08 |
| *Forage & Security* |  |  |  |  |  |  |  |  |
|  | Forbs + V cover + H cover + Dist edge + Dist edge^2^ + SSI |  | 7 | 46.8 | 35.0 | 64.2 | 2.5 | 0.14 |
|  | Berries + V cover + H cover + Dist edge + Dist edge^2^ + SSI |  | 7 | 49.2 | 31.7 | 66.6 | 4.9 | 0.04 |
|  | Ungulates + Dist edge + Dist edge^2^ + SSI |  | 5 | 55.3 | 23.3 | 67.0 | 5.3 | 0.03 |
|  | Forbs + Ungulates + Dist edge + Dist edge^2^ + SSI |  | 6 | 52.6 | 27.0 | 67.1 | 5.4 | 0.03 |

SSI - site severity index; LL - log likelihood; % Dev. explained - percentage deviance explained

**Table S3** Model structure and deviance for top 3 resting-site selection models (RSFs) for grizzly bear resting in protected areas in west-central Alberta, Canada. Model assessment was done by ranking AIC_c_ values (Δ*_i_*) and weights (*w_i_*) describing model likelihood. Model complexity (number of parameters) is given by *K_i_*. The top resting-site selection models were selected from candidate food, security and combined food and security models also selected via Δ*_i_* and *w_i_*. Only the top models from the latter categories are given below with the full set of models available in Table S8. The best overall model is given in bold.

|  | **Model** |  | ***K_i_*** | **-2LL** | **% Dev. explained** | **AIC_c_** | **Δ*_i_*** | ***w_i_*** |
| --- | --- | --- | --- | --- | --- | --- | --- | --- |
|  | Null model |  | 1 | 54.1 | 0.0 | 56.2 | 13.9 | 0.00 |
| *Forage* |  |  |  |  |  |  |  |  |
|  | Berries + SSI |  | 3 | 48.5 | 10.3 | 55.2 | 12.9 | 0.00 |
|  | Berries + Ungulates + SSI |  | 4 | 46.6 | 13.8 | 55.7 | 13.5 | 0.00 |
| *Security* |  |  |  |  |  |  |  |  |
|  | V cover + SSI |  | 3 | 35.8 | 33.8 | 42.5 | 0.2 | 0.33 |
|  | V cover + H cover + SSI |  | 4 | 35.1 | 35.2 | 44.2 | 1.9 | 0.14 |
|  | V cover + H cover + Dist trail + Dist trail^2^ + Dist edge + Dist edge^2^ + SSI |  | 8 | 26.2 | 51.5 | 46.7 | 4.5 | 0.04 |
| *Forage & Security* |  |  |  |  |  |  |  |  |
|  | **Berries + V cover + H cover + SSI** |  | **5** | **30.5** | **43.5** | **42.3** | **0.0** | **0.37** |
|  | Berries + V cover + H cover + Dist edge + Dist edge^2^ + SSI |  | 7 | 28.0 | 48.2 | 45.4 | 3.2 | 0.08 |
|  | Forbs + V cover + H cover + SSI |  | 5 | 34.8 | 35.6 | 46.5 | 4.3 | 0.04 |

SSI - site severity index; LL - log likelihood; % Dev. explained - percentage deviance explained

**Table S4** Model structure and deviance for top 3 resting-site selection models (RSFs) for grizzly bear resting on non-mined Crown (public) land in west-central Alberta, Canada. Model assessment was done by ranking AIC_c_ values (Δ*_i_*) and weights (*w_i_*) describing model likelihood. Model complexity (number of parameters) is given by *K_i_*. The top resting-site selection models were selected from candidate food, security and combined food and security models also selected via Δ*_i_* and *w_i_*. Only the top models from the latter categories are given below with the full set of models available in Table S9. The best overall model is given in bold.

|  | **Model** |  | ***K_i_*** | **-2LL** | **% Dev. explained** | **AIC_c_** | **Δ*_i_*** | ***w_i_*** |
| --- | --- | --- | --- | --- | --- | --- | --- | --- |
|  | Null model |  | 1 | 260.6 | 0.0 | 262.7 | 34.5 | 0.00 |
| *Forage* |  |  |  |  |  |  |  |  |
|  | Forbs + Berries + SSI |  | 4 | 247.8 | 4.9 | 256.9 | 28.7 | 0.00 |
|  | Forbs + Berries + Roots + Ungulates + SSI |  | 6 | 243.2 | 6.7 | 257.7 | 29.5 | 0.00 |
|  | Forbs + Ungulates + SSI |  | 4 | 249.8 | 4.1 | 258.9 | 30.7 | 0.00 |
| *Security* |  |  |  |  |  |  |  |  |
|  | V cover + H cover + Dist edge + Dist edge^2^ + SSI |  | 6 | 214.4 | 17.7 | 228.9 | 0.7 | 0.28 |
|  | V cover + H cover + Dist edge + Dist edge^2^ + Slope + Slope^2^ + SSI |  | 8 | 209.6 | 19.6 | 230.1 | 1.8 | 0.16 |
|  | V cover + H cover + Dist trail + Dist trail^2^ + Dist edge + Dist edge^2^ + SSI |  | 8 | 211.5 | 18.8 | 232.0 | 3.8 | 0.06 |
| *Forage & Security* |  |  |  |  |  |  |  |  |
|  | **Forbs + V cover + H cover + Dist edge + Dist edge^2^ + SSI** |  | **7** | **210.8** | **19.1** | **228.2** | **0.0** | **0.40** |
|  | Berries + V cover + H cover + Dist edge + Dist edge^2^ + SSI |  | 7 | 214.4 | 17.7 | 231.8 | 3.6 | 0.07 |
|  | Forbs + V cover + H cover + SSI |  | 5 | 221.4 | 15.0 | 233.1 | 4.9 | 0.03 |

SSI - site severity index; LL - log likelihood; % Dev. explained - percentage deviance explained

**Table S5** Model structure and deviance for candidate models for grizzly bear resting on reclaimed mines in west-central Alberta, Canada. Model assessment was done by ranking AIC_c_ values (Δ*_i_*) and weights (*w_i_*) describing model likelihood. Model complexity (number of parameters) is given by *K_i_*. The top resting-site selection models were selected from candidate food, security and combined food and security models also selected via Δ*_i_* and *w_i_*.

|  | **Model** |  | ***K_i_*** | **-2LL** | **% Dev. explained** | **AIC_c_** | **Δ*_i_*** | ***w_i_*** |
| --- | --- | --- | --- | --- | --- | --- | --- | --- |
|  | Null model |  | 1 | 72.1 | 0.0 | 74.2 | 3.0 | 0.08 |
| *Forage* |  |  |  |  |  |  |  |  |
|  | Forbs + SSI |  | 3 | 64.6 | 10.4 | 71.2 | 0.0 | 0.35 |
|  | Forbs + Berries + SSI |  | 4 | 64.2 | 11.0 | 73.3 | 2.1 | 0.13 |
|  | Forbs + Roots + SSI |  | 4 | 64.4 | 10.7 | 73.5 | 2.3 | 0.11 |
|  | Forbs + Ungulates + SSI |  | 4 | 64.4 | 10.7 | 73.5 | 2.3 | 0.11 |
|  | Berries + SSI |  | 3 | 67.1 | 6.9 | 73.8 | 2.6 | 0.10 |
|  | Ungulates + SSI |  | 3 | 69.0 | 4.3 | 75.6 | 4.4 | 0.04 |
|  | Berries + Ungulates + SSI |  | 4 | 66.6 | 7.7 | 75.7 | 4.5 | 0.04 |
|  | Roots + SSI |  | 3 | 69.4 | 3.7 | 76.1 | 4.9 | 0.03 |
|  | Forbs + Berries + Roots + Ungulates + SSI |  | 6 | 63.8 | 11.4 | 78.3 | 7.1 | 0.01 |
| *Security* |  |  |  |  |  |  |  |  |
|  | V cover + H cover + Dist trail + Dist trail^2^ + Dist edge + Dist edge^2^ + SSI |  | 8 | 41.0 | 43.1 | 61.5 | 0.0 | 0.59 |
|  | V cover + H cover + Dist edge + Dist edge^2^ + SSI |  | 6 | 49.1 | 31.9 | 63.6 | 2.1 | 0.21 |
|  | Dist edge + Dist edge^2^ + SSI |  | 4 | 56.1 | 22.2 | 65.2 | 3.6 | 0.10 |
|  | V cover + H cover + Dist road + Dist road^2^ + Dist edge + Dist edge^2^ + SSI |  | 8 | 45.6 | 36.7 | 66.1 | 4.6 | 0.06 |
|  | V cover + H cover + Dist edge + Dist edge^2^ + Slope + Slope^2^ + SSI |  | 8 | 47.2 | 34.5 | 67.7 | 6.1 | 0.03 |
|  | H cover + SSI |  | 3 | 63.9 | 11.3 | 70.5 | 9.0 | 0.01 |
|  | Elevation + Slope + Slope^2^ + SSI |  | 5 | 60.2 | 16.5 | 71.9 | 10.4 | 0.00 |
|  | V cover + H cover + SSI |  | 4 | 63.9 | 11.3 | 73.0 | 11.5 | 0.00 |
|  | V cover + H cover + Elevation + Slope + Slope^2^ + SSI |  | 7 | 56.5 | 21.6 | 73.9 | 12.4 | 0.00 |
|  | V cover + SSI |  | 3 | 67.5 | 6.4 | 74.1 | 12.6 | 0.00 |
|  | V cover + H cover + Dist trail + Dist trail^2^ + Slope + Slope^2^ + SSI |  | 8 | 55.0 | 23.7 | 75.5 | 13.9 | 0.00 |
|  | V cover + H cover + Dist road + Dist road^2^ + Slope + Slope^2^ + SSI |  | 8 | 56.7 | 21.4 | 77.2 | 15.6 | 0.00 |
|  | V cover + H cover + Dist road + Dist road^2^ + Dist trail + Distr trail^2^ + SSI |  | 8 | 62.2 | 13.7 | 82.7 | 21.2 | 0.00 |
|  | Dist road + Dist road^2^ + Dist trail + Dist trail^2^ + SSI |  | 6 | 68.8 | 4.6 | 83.3 | 21.7 | 0.00 |
| *Forage & Security* |  |  |  |  |  |  |  |  |
|  | Forbs + V cover + H cover + Dist edge + Dist edge^2^ + SSI |  | 7 | 47.0 | 34.9 | 64.4 | 0.0 | 0.53 |
|  | Berries + V cover + H cover + Dist edge + Dist edge^2^ + SSI |  | 7 | 49.1 | 31.9 | 66.5 | 2.2 | 0.18 |
|  | Ungulates + Dist edge + Dist edge^2^ + SSI |  | 5 | 55.2 | 23.5 | 66.9 | 2.5 | 0.15 |
|  | Forbs + Ungulates + Dist edge + Dist edge^2^ + SSI |  | 6 | 53.1 | 26.4 | 67.5 | 3.2 | 0.11 |
|  | Forbs + V cover + H cover + SSI |  | 5 | 60.7 | 15.8 | 72.4 | 8.1 | 0.01 |
|  | Roots + V cover + H cover + Slope + Slope^2^ + SSI |  | 7 | 55.3 | 23.3 | 72.7 | 8.3 | 0.01 |

**Table S5 Continued**

|  | **Model** |  | ***K_i_*** | **-2LL** | **% Dev. explained** | **AIC_c_** | **Δ*_i_*** | ***w_i_*** |
| --- | --- | --- | --- | --- | --- | --- | --- | --- |
|  | Ungulates + V cover + H cover + Slope + Slope^2^ + SSI |  | 7 | 56.2 | 22.1 | 73.6 | 9.2 | 0.01 |
|  | Berries + V cover + H cover + SSI |  | 5 | 63.0 | 12.5 | 74.8 | 10.4 | 0.00 |
|  | Roots + V cover + H cover + SSI |  | 5 | 63.1 | 12.4 | 74.9 | 10.5 | 0.00 |
|  | Forbs + Dist road + Dist road^2^ + Dist trail + Dist trail^2^ + SSI |  | 7 | 62.4 | 13.4 | 79.8 | 15.5 | 0.00 |
|  | Forbs + Berries + Roots + Ungulates + V cover + H cover + SSI |  | 8 | 59.7 | 17.2 | 80.2 | 15.8 | 0.00 |
|  | Forbs + Ungulates + Dist road + Dist road^2^ + Dist trail + Dist trail^2^ + SSI |  | 8 | 62.1 | 13.8 | 82.6 | 18.3 | 0.00 |
|  | Ungulates + Dist road + Dist road^2^ + Dist trail + Dist trail^2^ + SSI |  | 7 | 68.2 | 5.4 | 85.6 | 21.3 | 0.00 |

% Dev. explained - percentage deviance explained

**Table S6** Model structure and deviance for candidate models for grizzly bear resting in protected areas in west-central Alberta, Canada. Model assessment was done by ranking AIC_c_ values (Δ*_i_*) and weights (*w_i_*) describing model likelihood. Model complexity (number of parameters) is given by *K_i_*. The top bedding site selection models were selected from candidate food, security and combined food and security models also selected via Δ*_i_* and *w_i_*.

|  | **Model** |  | ***K_i_*** | **-2LL** | **% Dev. explained** | **AIC_c_** | **Δ*_i_*** | ***w_i_*** |
| --- | --- | --- | --- | --- | --- | --- | --- | --- |
|  | Null model |  | 1 | 54.1 | 0.0 | 56.2 | 1.0 | 0.15 |
| *Forage* |  |  |  |  |  |  |  |  |
|  | Berries + SSI |  | 3 | 48.5 | 10.2 | 55.2 | 0.0 | 0.25 |
|  | Berries + Ungulates + SSI |  | 4 | 46.6 | 13.8 | 55.7 | 0.5 | 0.19 |
|  | Forbs + Berries + SSI |  | 4 | 47.1 | 12.9 | 56.2 | 1.0 | 0.15 |
|  | Forbs + Berries + Roots + Ungulates + SSI |  | 6 | 42.6 | 21.1 | 57.1 | 1.9 | 0.09 |
|  | Ungulates + SSI |  | 3 | 51.2 | 5.4 | 57.8 | 2.6 | 0.07 |
|  | Forbs + SSI |  | 3 | 52.4 | 3.0 | 59.1 | 3.9 | 0.04 |
|  | Roots + SSI |  | 3 | 53.0 | 2.0 | 59.6 | 4.4 | 0.03 |
|  | Forbs + Ungulates + SSI |  | 4 | 50.6 | 6.4 | 59.7 | 4.5 | 0.03 |
|  | Forbs + Roots + SSI |  | 4 | 51.5 | 4.7 | 60.7 | 5.5 | 0.02 |
| *Security* |  |  |  |  |  |  |  |  |
|  | V cover + SSI |  | 3 | 35.8 | 33.7 | 42.5 | 0.0 | 0.57 |
|  | V cover + H cover + SSI |  | 4 | 35.1 | 35.1 | 44.2 | 1.7 | 0.24 |
|  | V cover + H cover + Dist trail + Dist trail^2^ + Dist edge + Dist edge^2^ + SSI |  | 8 | 26.2 | 51.5 | 46.7 | 4.3 | 0.07 |
|  | V cover + H cover + Dist edge + Dist edge^2^ + SSI |  | 6 | 33.5 | 38.0 | 48.0 | 5.5 | 0.04 |
|  | V cover + H cover + Dist trail + Dist trail^2^ + Slope + Slope^2^ + SSI |  | 8 | 27.7 | 48.7 | 48.2 | 5.8 | 0.03 |
|  | V cover + H cover + Dist road + Dist road^2^ + Dist trail + Distr trail^2^ + SSI |  | 8 | 27.8 | 48.6 | 48.3 | 5.8 | 0.03 |
|  | V cover + H cover + Elevation + Slope + Slope^2^ + SSI |  | 7 | 34.8 | 35.6 | 52.2 | 9.8 | 0.00 |
|  | H cover + SSI |  | 3 | 45.9 | 15.1 | 52.5 | 10.1 | 0.00 |
|  | V cover + H cover + Dist road + Dist road^2^ + Dist edge + Dist edge^2^ + SSI |  | 8 | 33.1 | 38.8 | 53.6 | 11.1 | 0.00 |
|  | V cover + H cover + Dist edge + Dist edge^2^ + Slope + Slope^2^ + SSI |  | 8 | 33.5 | 38.1 | 54.0 | 11.5 | 0.00 |
|  | V cover + H cover + Dist road + Dist road^2^ + Slope + Slope^2^ + SSI |  | 8 | 34.8 | 35.6 | 55.3 | 12.9 | 0.00 |
|  | Dist edge + Dist edge^2^ + SSI |  | 4 | 51.0 | 5.7 | 60.1 | 17.6 | 0.00 |
|  | Dist road + Dist road^2^ + Dist trail + Dist trail^2^ + SSI |  | 6 | 50.0 | 7.5 | 64.5 | 22.0 | 0.00 |
|  | Elevation + Slope + Slope^2^ + SSI |  | 5 | 53.0 | 2.0 | 64.7 | 22.2 | 0.00 |
| *Forage & Security* |  |  |  |  |  |  |  |  |
|  | Berries + V cover + H cover + SSI |  | 5 | 30.5 | 43.5 | 42.3 | 0.0 | 0.58 |
|  | Forbs + V cover + H cover + SSI |  | 5 | 33.2 | 38.6 | 44.9 | 2.6 | 0.15 |
|  | Berries + V cover + H cover + Dist edge + Dist edge^2^ + SSI |  | 7 | 28.0 | 48.1 | 45.4 | 3.2 | 0.12 |
|  | Forbs + Berries + Roots + Ungulates + V cover + H cover + SSI |  | 8 | 26.1 | 51.7 | 46.6 | 4.4 | 0.07 |
|  | Roots + V cover + H cover + SSI |  | 5 | 35.0 | 35.3 | 46.7 | 4.5 | 0.06 |
|  | Forbs + V cover + H cover + Dist edge + Dist edge^2^ + SSI |  | 7 | 32.6 | 39.7 | 50.0 | 7.7 | 0.01 |

**Table S6 Continued**

|  | **Model** |  | ***K_i_*** | **-2LL** | **% Dev. explained** | **AIC_c_** | **Δ*_i_*** | ***w_i_*** |
| --- | --- | --- | --- | --- | --- | --- | --- | --- |
|  | Ungulates + V cover + H cover + Slope + Slope^2^ + SSI |  | 7 | 34.6 | 36.1 | 52.0 | 9.7 | 0.00 |
|  | Roots + V cover + H cover + Slope + Slope^2^ + SSI |  | 7 | 34.7 | 35.7 | 52.1 | 9.9 | 0.00 |
|  | Ungulates + Dist edge + Dist edge^2^ + SSI |  | 5 | 49.3 | 8.7 | 61.1 | 18.8 | 0.00 |
|  | Forbs + Ungulates + Dist edge + Dist edge^2^ + SSI |  | 6 | 49.0 | 9.4 | 63.5 | 21.2 | 0.00 |
|  | Ungulates + Dist road + Dist road^2^ + Dist trail + Dist trail^2^ + SSI |  | 7 | 47.1 | 12.9 | 64.5 | 22.2 | 0.00 |
|  | Forbs + Dist road + Dist road^2^ + Dist trail + Dist trail^2^ + SSI |  | 7 | 49.3 | 8.8 | 66.7 | 24.5 | 0.00 |
|  | Forbs + Ungulates + Dist road + Dist road^2^ + Dist trail + Dist trail^2^ + SSI |  | 8 | 46.7 | 13.7 | 67.2 | 24.9 | 0.00 |

% Dev. explained - percentage deviance explained

**Table S7** Model structure and deviance for candidate models for grizzly bear resting on non-mined Crown (public) land in west-central Alberta, Canada. Model assessment was done by ranking AIC_c_ values (Δ*_i_*) and weights (*w_i_*) describing model likelihood. Model complexity (number of parameters) is given by *K_i_*. The top bedding site selection models were selected from candidate food, security and combined food and security models also selected via Δ*_i_* and *w_i_*.

|  | **Model** |  | ***K_i_*** | **-2LL** | **% Dev. explained** | **AIC_c_** | **Δ*_i_*** | ***w_i_*** |
| --- | --- | --- | --- | --- | --- | --- | --- | --- |
|  | Null model |  | 1 | 260.6 | 0.0 | 262.7 | 2.3 | 0.06 |
| *Forage* |  |  |  |  |  |  |  |  |
|  | Forbs + Berries + SSI |  | 4 | 251.3 | 3.6 | 260.4 | 0.0 | 0.20 |
|  | Berries + Ungulates + SSI |  | 4 | 251.5 | 3.5 | 260.7 | 0.2 | 0.18 |
|  | Forbs + Ungulates + SSI |  | 4 | 251.7 | 3.4 | 260.8 | 0.4 | 0.16 |
|  | Forbs + Berries + Roots + Ungulates + SSI |  | 6 | 246.8 | 5.3 | 261.2 | 0.8 | 0.13 |
|  | Forbs + SSI |  | 3 | 255.3 | 2.0 | 262.0 | 1.6 | 0.09 |
|  | Berries + SSI |  | 3 | 255.7 | 1.9 | 262.3 | 1.9 | 0.08 |
|  | Ungulates + SSI |  | 3 | 256.5 | 1.6 | 263.1 | 2.7 | 0.05 |
|  | Forbs + Roots + SSI |  | 4 | 254.8 | 2.2 | 263.9 | 3.5 | 0.04 |
|  | Roots + SSI |  | 3 | 259.8 | 0.3 | 266.4 | 6.0 | 0.01 |
| *Security* |  |  |  |  |  |  |  |  |
|  | V cover + H cover + Dist edge + Dist edge^2^ + SSI |  | 6 | 214.3 | 17.8 | 228.8 | 0.0 | 0.52 |
|  | V cover + H cover + Dist edge + Dist edge^2^ + Slope + Slope^2^ + SSI |  | 8 | 209.5 | 19.6 | 230.0 | 1.2 | 0.28 |
|  | V cover + H cover + Dist trail + Dist trail^2^ + Dist edge + Dist edge^2^ + SSI |  | 8 | 211.4 | 18.9 | 231.9 | 3.1 | 0.11 |
|  | V cover + H cover + Dist road + Dist road^2^ + Dist edge + Dist edge^2^ + SSI |  | 8 | 212.1 | 18.6 | 232.6 | 3.8 | 0.08 |
|  | V cover + H cover + SSI |  | 4 | 226.9 | 12.9 | 236.0 | 7.2 | 0.01 |
|  | V cover + H cover + Elevation + Slope + Slope^2^ + SSI |  | 7 | 224.5 | 13.9 | 241.9 | 13.1 | 0.00 |
|  | V cover + H cover + Dist road + Dist road^2^ + Dist trail + Distr trail^2^ + SSI |  | 8 | 221.9 | 14.8 | 242.4 | 13.6 | 0.00 |
|  | V cover + H cover + Dist trail + Dist trail^2^ + Slope + Slope^2^ + SSI |  | 8 | 223.4 | 14.3 | 243.9 | 15.1 | 0.00 |
|  | V cover + H cover + Dist road + Dist road^2^ + Slope + Slope^2^ + SSI |  | 8 | 223.5 | 14.2 | 244.0 | 15.2 | 0.00 |
|  | V cover + SSI |  | 3 | 237.9 | 8.7 | 244.5 | 15.7 | 0.00 |
|  | H cover + SSI |  | 3 | 244.4 | 6.2 | 251.0 | 22.2 | 0.00 |
|  | Elevation + Slope + Slope^2^ + SSI |  | 5 | 255.8 | 1.8 | 267.5 | 28.7 | 0.00 |
|  | Dist edge + Dist edge^2^ + SSI |  | 4 | 249.3 | 4.3 | 258.4 | 29.6 | 0.00 |
|  | Dist road + Dist road^2^ + Dist trail + Dist trail^2^ + SSI |  | 6 | 253.4 | 2.8 | 267.9 | 39.1 | 0.00 |
| *Forage & Security* |  |  |  |  |  |  |  |  |
|  | Forbs + V cover + H cover + Dist edge + Dist edge^2^ + SSI |  | 7 | 211.7 | 18.8 | 229.1 | 0.0 | 0.67 |
|  | Berries + V cover + H cover + Dist edge + Dist edge^2^ + SSI |  | 7 | 214.3 | 17.8 | 231.7 | 2.6 | 0.19 |
|  | Forbs + V cover + H cover + SSI |  | 5 | 221.4 | 15.1 | 233.1 | 4.0 | 0.09 |
|  | Forbs + Berries + Roots + Ungulates + V cover + H cover + SSI |  | 8 | 215.7 | 17.2 | 236.2 | 7.0 | 0.02 |
|  | Roots + V cover + H cover + SSI |  | 5 | 225.2 | 13.6 | 236.9 | 7.8 | 0.01 |
|  | Berries + V cover + H cover + SSI |  | 5 | 226.8 | 13.0 | 238.6 | 9.4 | 0.01 |

**Table S7** Continued

|  | **Model** |  | ***K_i_*** | **-2LL** | **% Dev. explained** | **AIC_c_** | **Δ*_i_*** | ***w_i_*** |
| --- | --- | --- | --- | --- | --- | --- | --- | --- |
|  | Ungulates + V cover + H cover + Slope + Slope^2^ + SSI |  | 7 | 221.7 | 14.9 | 239.1 | 9.9 | 0.00 |
|  | Roots + V cover + H cover + Slope + Slope^2^ + SSI |  | 7 | 223.9 | 14.1 | 241.3 | 12.2 | 0.00 |
|  | Ungulates + Dist edge + Dist edge^2^ + SSI |  | 5 | 246.0 | 5.6 | 257.7 | 28.6 | 0.00 |
|  | Forbs + Ungulates + Dist edge + Dist edge^2^ + SSI |  | 6 | 243.7 | 6.5 | 258.2 | 29.0 | 0.00 |
|  | Forbs + Ungulates + Dist road + Dist road^2^ + Dist trail + Dist trail^2^ + SSI |  | 8 | 243.8 | 6.5 | 264.3 | 35.1 | 0.00 |
|  | Ungulates + Dist road + Dist road^2^ + Dist trail + Dist trail^2^ + SSI |  | 7 | 248.1 | 4.8 | 265.5 | 36.4 | 0.00 |
|  | Forbs + Dist road + Dist road^2^ + Dist trail + Dist trail^2^ + SSI |  | 7 | 248.4 | 4.7 | 265.8 | 36.7 | 0.00 |

% Dev. explained - percentage deviance explained

**Table S8** Model structure and deviance for top GLM models testing the influence of season, land designation and time of day on selection ratios for grizzly bear resting in west-central Alberta, Canada. Model assessment was done by ranking AIC_c_ values (Δ*_i_*) and weights (*w_i_*) describing model likelihood. Model complexity (number of parameters) is given by *K_i_*.

| **Model** | ***K_i_*** | **-2LL** | **% Dev. explained** | **AIC_c_** | **Δ*_i_*** | ***w_i_*** |
| --- | --- | --- | --- | --- | --- | --- |
| *V cover selection ratio* |  |  |  |  |  |  |
| Null model | 1 | 1351.9 | 0.0 | 1354.0 | 23.1 | 0.00 |
| Land designation + Season + Land designation × Season | 4 | 1321.9 | 10.2 | 1331.0 | 0.0 | 1.00 |
| *H cover selection ratio* |  |  |  |  |  |  |
| Null model | 1 | 627.8 | 0.0 | 629.9 | 15.3 | 0.00 |
| Land designation + Season + Land designation × Season | 4 | 605.6 | 7.7 | 614.7 | 0.0 | 0.99 |
| *Dist to edge selection ratio* |  |  |  |  |  |  |
| Null model | 1 | 1088.9 | 0.0 | 1091.0 | 22.2 | 0.00 |
| Land designation + Time of day + Land designation × Time of day | 4 | 1059.7 | 9.9 | 1068.8 | 0.0 | 0.49 |
| *Dist to trail selection ratio* |  |  |  |  |  |  |
| Null model | 1 | 992.3 | 0.0 | 994.4 | 0.3 | 0.19 |
| Land designation | 2 | 989.8 | 0.9 | 994.1 | 0.0 | 0.22 |

All dependent variables were log-transformed to create a Gaussian distribution

LL - log likelihood; % Dev. explained - percentage deviance explained

**Table S9** Model structure, deviance, significance and goodness-of-fit (Wald _χ_^2^) for top GLM models testing the influence of season, land designation and time of day on vertical and horizontal cover selection ratios for grizzly bear resting in west-central Alberta, Canada. Model assessment was done by ranking AIC_c_ values (Δ*_i_*) and weights (*w_i_*) describing model likelihood. Model complexity (number of parameters) is given by *K_i_*. The full set of candidate models including the null models is provided below.

| **Model** | ***K_i_*** | **-2LL** | **% Dev. explained** | **AIC_c_** | **Δ*_i_*** | ***w_i_*** |
| --- | --- | --- | --- | --- | --- | --- |
| *V cover selection ratio* |  |  |  |  |  |  |
| Null model | 1 | 1351.9 | 0.0 | 1354.0 | 23.1 | 0.00 |
| Land designation + Season + Land designation × Season | 4 | 1321.9 | 10.2 | 1331.0 | 0.0 | 1.00 |
| Land designation + Time of day + Land designation × Time of day | 4 | 1335.4 | 5.8 | 1344.5 | 13.5 | 0.00 |
| Season + Land designation | 3 | 1339.9 | 4.2 | 1346.5 | 15.6 | 0.00 |
| Land designation | 2 | 1343.3 | 3.0 | 1347.6 | 16.6 | 0.00 |
| Season + Land designation + Time of day | 4 | 1338.6 | 4.6 | 1347.8 | 16.8 | 0.00 |
| Land designation + Time of day | 3 | 1342.1 | 3.5 | 1348.7 | 17.8 | 0.00 |
| Season + Time of day + Season × Time of day | 4 | 1342.6 | 3.3 | 1351.7 | 20.7 | 0.00 |
| Season + Time of day | 3 | 1345.1 | 2.4 | 1351.8 | 20.8 | 0.00 |
| Season | 2 | 1347.6 | 1.5 | 1351.9 | 21.0 | 0.00 |
| Time of day | 2 | 1349.5 | 0.9 | 1253.8 | 22.8 | 0.00 |
| *H cover selection ratio* |  |  |  |  |  |  |
| Null model | 1 | 627.8 | 0.0 | 629.9 | 15.3 | 0.00 |
| Land designation + Season + Land designation × Season | 4 | 605.6 | 7.7 | 614.7 | 0.0 | 0.99 |
| Season + Time of day + Season × Time of day | 4 | 615.7 | 4.3 | 624.8 | 10.2 | 0.01 |
| Land designation | 2 | 625.9 | 0.6 | 630.2 | 15.5 | 0.00 |
| Season | 2 | 626.1 | 0.6 | 630.4 | 15.8 | 0.00 |
| Season + Land designation | 3 | 624.1 | 1.3 | 630.7 | 16.1 | 0.00 |
| Land designation + Time of day + Land designation × Time of day | 4 | 622.7 | 1.8 | 631.9 | 17.2 | 0.00 |
| Time of day | 2 | 627.7 | 0.0 | 632.0 | 17.4 | 0.00 |
| Land designation + Time of day | 3 | 625.5 | 0.8 | 632.1 | 17.5 | 0.00 |
| Season + Time of day | 3 | 626.1 | 0.6 | 632.7 | 18.1 | 0.00 |
| Season + Land designation + Time of day | 4 | 623.9 | 1.4 | 633.0 | 18.3 | 0.00 |
